# A single incidental dark pulse during daytime attenuated food anticipatory behavior

**DOI:** 10.1080/19420889.2024.2341050

**Published:** 2024-04-23

**Authors:** Khaviya Balaji, S. K. Tahajjul Taufique, Melody Shen, David E. Ehichioya, Sofia Farah, Shin Yamazaki

**Affiliations:** a Department of Neuroscience, UT Southwestern Medical Center, Dallas, TX, USA; b Wakeland High School, Frisco, TX, USA; c Peter O’Donnell Jr. Brain Institute, UT Southwestern Medical Center, Dallas, TX, USA

**Keywords:** Circadian rhythm, extra-SCN pacemaker, food anticipation, food seeking behavior, light entrainment, non-canonical circadian oscillator, operant, reward

## Abstract

Using an open-source operant feeding device (FED3), we measured food-seeking nose poking behavior in mice. When the mice were exposed to 4 h restricted feeding at night, all mice exhibited robust food anticipatory nose poking starting ~4 h before scheduled mealtime. When the light-dark cycle was advanced by 6 h, mice exhibited two distinct bouts of anticipatory poking, one corresponding to actual mealtime which continued at the same time of day, and one corresponding to predicted mealtime which shifted parallel with the light-dark cycle. Likewise, two similar bouts of food-seeking behavior appeared when the light-dark cycle was delayed for 9 h. These data suggest that food anticipatory behavior is encoded to a circadian oscillator that entrains to the light-dark cycle. Two weeks after advancing the light-dark cycle, mice incidentally received a 3.5 h dark pulse in the middle of the day. This single dark pulse had a negligible effect on running wheel behavior but caused a temporary attenuation of both food anticipatory poking and pellet intake. These results suggest that the circadian oscillator controlling food anticipatory poking is sensitive to light disruption and that proper food anticipation is critical for sufficient food intake during temporally restricted feeding.

Anticipation of daily changes in the environment is critical for the fitness of living organisms. The body’s internal time-keeping mechanism enables the organisms to anticipate predictable daily changes in the environment, such as daily food availability [1,2]. It has been well demonstrated in rodents that time-restricted food availability can elicit arousal and anticipatory activity several hours before daily mealtime [3]. This food anticipatory activity (FAA) is controlled by a putative food-entrainable oscillator (FEO) which is located outside of the primary circadian pacemaker in the suprachiasmatic nucleus (SCN) [4] and independent from the well-defined circadian molecular transcriptional-translational feedback loop [5]. Although FAA was first described over 100 years ago [3] and the characteristics of the FEO have been described for over 50 years [1,2], the location and molecular mechanism of the FEO still remained elusive [6,7].

Recently, we used the operant feeding device, the Feeding Experimentation Device version 3 (FED3) [8], and made several observations that are consistent with the novel hypothesis that the time of food availability is encoded on a light sensitive circadian oscillator located outside of the SCN [9]. In that study, we programmed the FED3 to dispense pellets based on poking of the nose-poke holes, with poking of the left nose-poke hole as rewarded food-seeking behavior and poking of the right nose-poke hole as unrewarded food-seeking behavior [10]. Both rewarded and unrewarded anticipatory pokes for daytime restricted meals shifted along with the light-dark cycle, even though we kept the feeding schedule unchanged. In the current study, mice underwent the same protocol, except we shifted the environmental light-dark cycle while mice were under 4 h restricted feeding at night. While we were performing this experiment, mice received an incidental 3.5 h dark pulse in the middle of the day. Unexpectedly, this one-time short dark pulse significantly disrupted both anticipatory nose pokes and pellet intake. This result further supports our conclusion that mice use a light-entrainable oscillator to anticipate food availability.

All experiments were carried out in accordance with the National Institutes of Health Guidelines regarding the care and use of animals for experimental procedures and were approved by the Institutional Animal Care and Use Committee at UT Southwestern Medical Center (Protocol #: 2016–10376-G). 103-to-169-day-old C57BL/6N male mice (*n* = 5) were individually housed in small running wheel cages (32.5 cm in length, 14.5 cm in width, and 13 cm in height, equipped with 11 cm diameter running wheel). FED3 was attached to the outside of the cage (mice had access to the pellet dispenser hole and nose-poke holes through an ~8 × 3 cm opening on the side of the cage). After 7 days of *ad libitum* feeding, mice were transitioned to 4 h restricted feeding at night by gradually reducing food availability. Within a few days, the mice started showing consolidated anticipatory nose poking ~4 h before the onset of feeding time (Figures 1, 2, and S1). This anticipatory poking was stronger in rewarded left nose pokes compared with unrewarded right nose pokes (Figures 1, 2, and S1). FAA could not be detected, as it occurred during the dark (active) phase and was difficult to differentiate from SCN-controlled nocturnal activity (Figure 1 and S1, S2). However, there was a notable ~1 h break in wheel running activity at the beginning of food availability. We next advanced the light-dark cycle by 6 h while keeping the feeding time unchanged. In addition to the anticipatory pokes at the unchanged feeding time, a distinct bout of poking, corresponding to the mealtime if it had shifted parallel with the light-dark cycle, also appeared. Therefore, mice exhibited two bouts of anticipatory poking, one for the current, unchanged feeding time, and one for the previous feeding schedule, which shifted in tandem with the light-dark cycle to remain in the middle of the night (Figures 1, 2, and S1). In our previous study, we didn’t observe two bouts of anticipatory poking when we advanced the light-dark cycle while mice were fed during the day. It has been shown that the SCN phase dependently inhibits FAA [11]. This may contribute to the difference between current and previous studies. Thereafter, we delayed the light-dark cycle by 9 h while keeping the feeding time unchanged. The onset of anticipatory nose pokes was rapidly delayed, and the mice showed two distinct, separate bouts of anticipatory poking (Figures 1, 2, and S1). This is consistent with our previous report [9].

**Figure 1.**
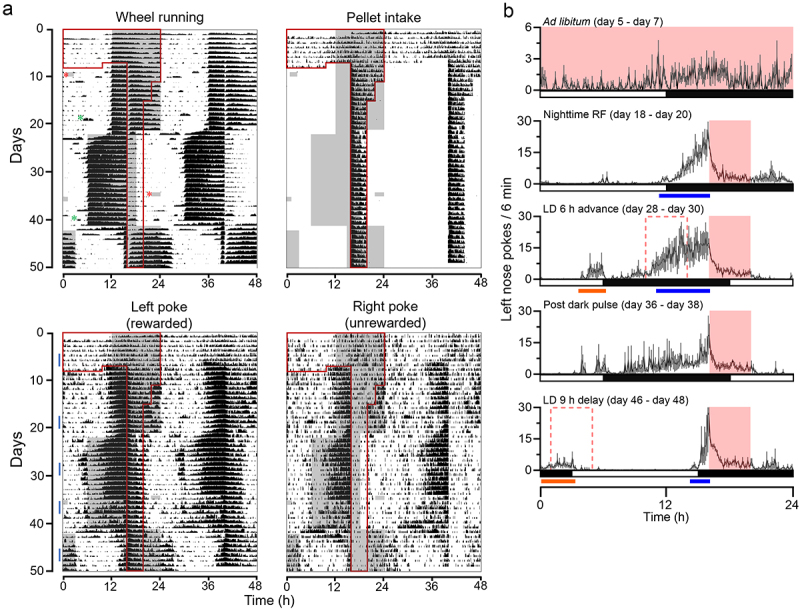
Food availability time is encoded to the light-entrainable circadian oscillator. (A) Average double-plotted ethograms for wheel running activity, pellet intake, left (rewarded) nose pokes, and right (unrewarded) nose pokes, recorded from C57BL/6N male mice (*n* = 5). Data was plotted percentile plot in 6 min bins, quantiles of 50. Individual ethograms of all male mice are shown in Figure S1. On the left half of the double-plotted ethograms, night (dark) is indicated as grey shading, and the time of food availability is outlined in red lines. Due to a technical issue (forced Windows update), the mice received two incidental dark pulses, a 2 h dark pulse at the beginning of day 9 and a 3.5 h dark pulse in the middle of the day on day 35. These are indicated with red asterisks in the Wheel Running subpanel. This incidence did not affect the data collection by FED3, as it is battery-operated. Cage changes are indicated by green asterisks. (B) Group average 24-h profiles of the left nose pokes of 3 days during *ad libitum* (day 5 – day 7), 3 days during night-time restricted feeding (day 18 – day 20), 3 days during the period after the light-dark cycle was advanced (day 28 – day 30), 3 days immediately after the incidental dark pulse (day 36 – day 38), and 3 days during the period after the light-dark cycle was delayed (day 46 – day 48). The time of food availability is indicated by the solid pink box. The pink dotted box indicates the time of food availability if it were linked to the phase of the light-entrainable oscillator. Data are presented as mean ±SEM. Blue bars indicate bouts of food anticipatory poking for current mealtime. Orange bars indicate bouts of phase-shifted food anticipatory poking for previous mealtime.

**Figure 2.**
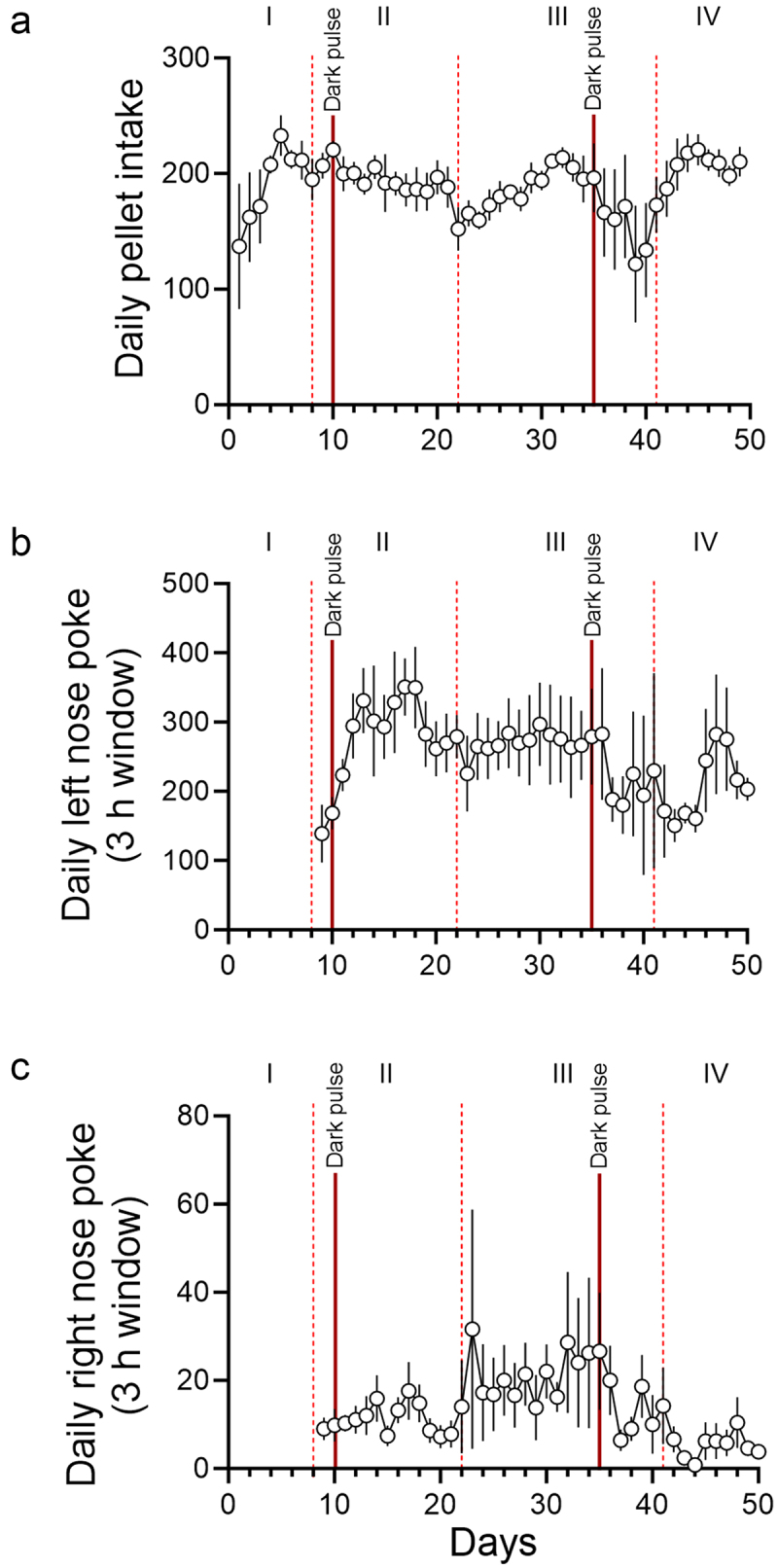
Daily changes in pellet intake, anticipatory rewarded left nose pokes, and anticipatory unrewarded right nose pokes. Daily pellet intake (pellets/day; A), anticipatory rewarded left nose pokes 3 h before the feeding schedule (pokes/3 h window; B), and anticipatory unrewarded right nose pokes 3 h before the feeding schedule (pokes/3 h window; C) are shown. The days in which light schedules were changed are indicated with vertical dotted lines. The days when incidental dark pulses were given to the mice are indicated with solid red lines. Roman numerals (I: *ad libitum* feeding, II: nighttime restricted feeding, III: 6 h light-dark cycle advance, IV: 9 h light-dark cycle delay). Data are presented as mean ±SEM (*n* = 5).

Due to a forced Windows update, mice received two incidental dark pulses. The first dark pulse was 2 h on day 10 at the beginning of the day. This dark pulse didn’t cause a notable change in either the wheel running activity or the food intake. In contrast, the second 3.5 h dark pulse in the middle of the day on day 35 had an unexpectedly strong effect on both food anticipatory poking and food intake. In the following days, some mice failed to anticipate food availability (Figure 1 and S1). Analysis of the anticipatory nose-pokes and pellet intake revealed that, immediately following the day of the dark pulse, both left and right nose-pokes were attenuated, and pellet intake was decreased (Figure 2). We interpreted this disruption as the single dark pulse acutely shifting the phase of the extra-SCN circadian pacemaker on which mealtime is encoded, causing mice to be unable to anticipate mealtime until the pacemaker re-entrained to the environmental light-dark cycle. Thus, these data further support the hypothesis that mice use a light-sensitive circadian oscillator to anticipate mealtime. This incidental dark pulse also implicates the ecological significance of food anticipation. There was a positive correlation between anticipatory poking and pellet intake (Figure S3), suggesting that proper anticipation of food is critical for sufficient energy intake when access to food is temporally restricted. Furthermore, the SCN-controlled wheel running activity was, contrastingly, largely unaffected by the dark pulse. This is consistent with the consensus in the field that the light-entrainable circadian oscillator which controls food anticipation is not in the SCN [4,7]. The difference in the effects of the two dark pulses may be explained by the difference in the time of each dark pulse. It is well-known that a dark pulse during the middle of the day has a stronger effect on the circadian behavior activity rhythm than a dark pulse at another time [12]. The effect of a dark pulse on the extra-SCN light entrainable circadian pacemaker likely has a similar difference in effect based on time of day. As the first dark pulse occurred early in the light phase, this may be the reason why the first incidental dark pulse had a minimal effect on anticipatory poking and pellet intake, as opposed to the second dark pulse, which occurred closer to the middle of the light phase.

The current study highlights the light sensitive nature of the FEO, which controls food anticipatory behavior, and that proper food anticipation is critical for sufficient energy intake when food availability becomes limited to a few hours per day.

## Supplementary Material

Supplemental Material

## Data Availability

Data reported in this paper is available upon request.
