# Supplementary material for: A single incidental dark pulse during daytime attenuated food anticipatory behavior
Source: Commun Integr Biol. 2024 Apr 23;17(1):2341050. doi: 10.1080/19420889.2024.2341050 (PMC11057643; doi:10.1080/19420889.2024.2341050)
Supplement: Supplemental Material [file KCIB_A_2341050_SM9723.docx]

**Supplementary material**


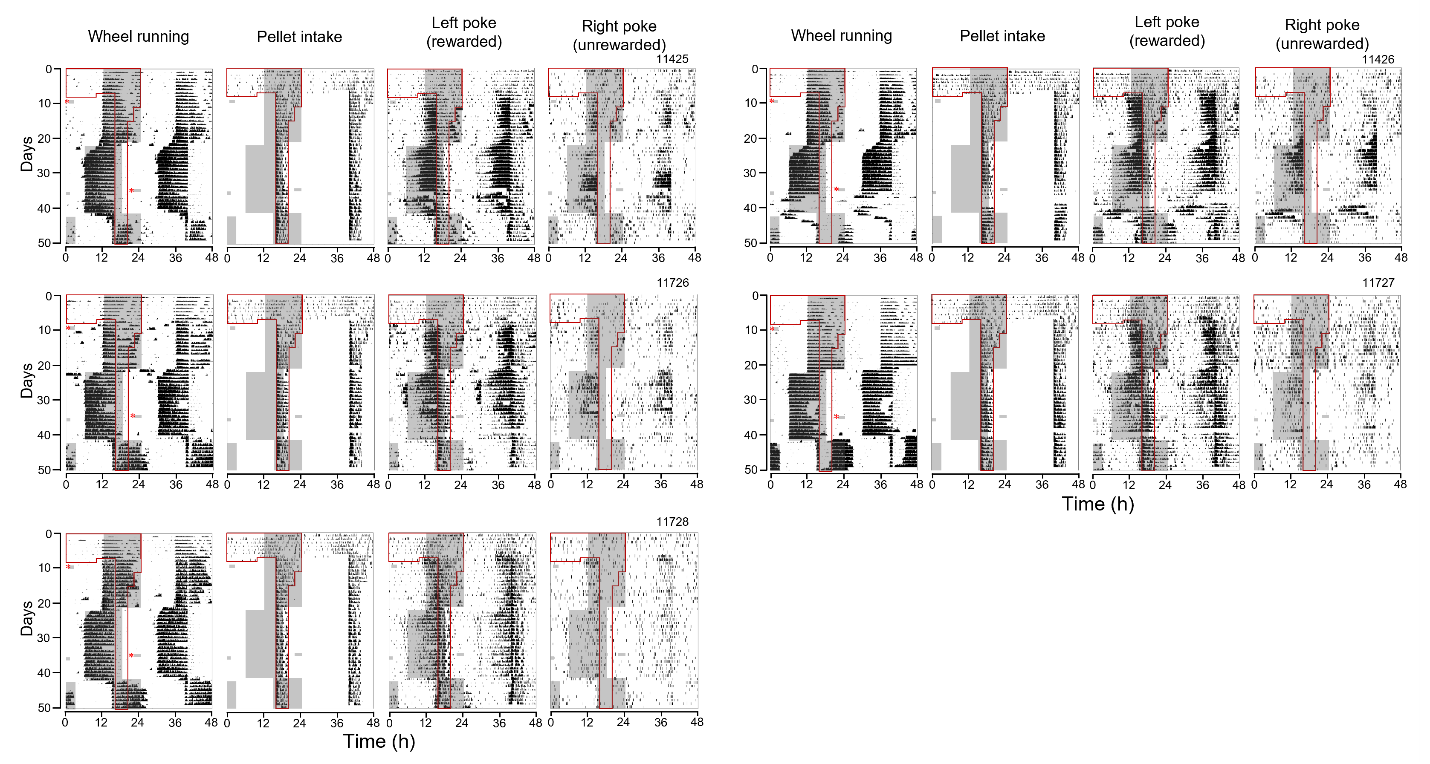


**Supplementary Figure 1: Double plotted ethograms for wheel running activity, pellet intake, left nose poke, and right nose poke of all 5 individual C67BL/6N male mice.** Related to Figure 1. On the left half of the individual double-plotted ethograms, night (dark) is indicated as grey shading, and the time of food availability is outlined in red lines. See details in Figure 1.

**
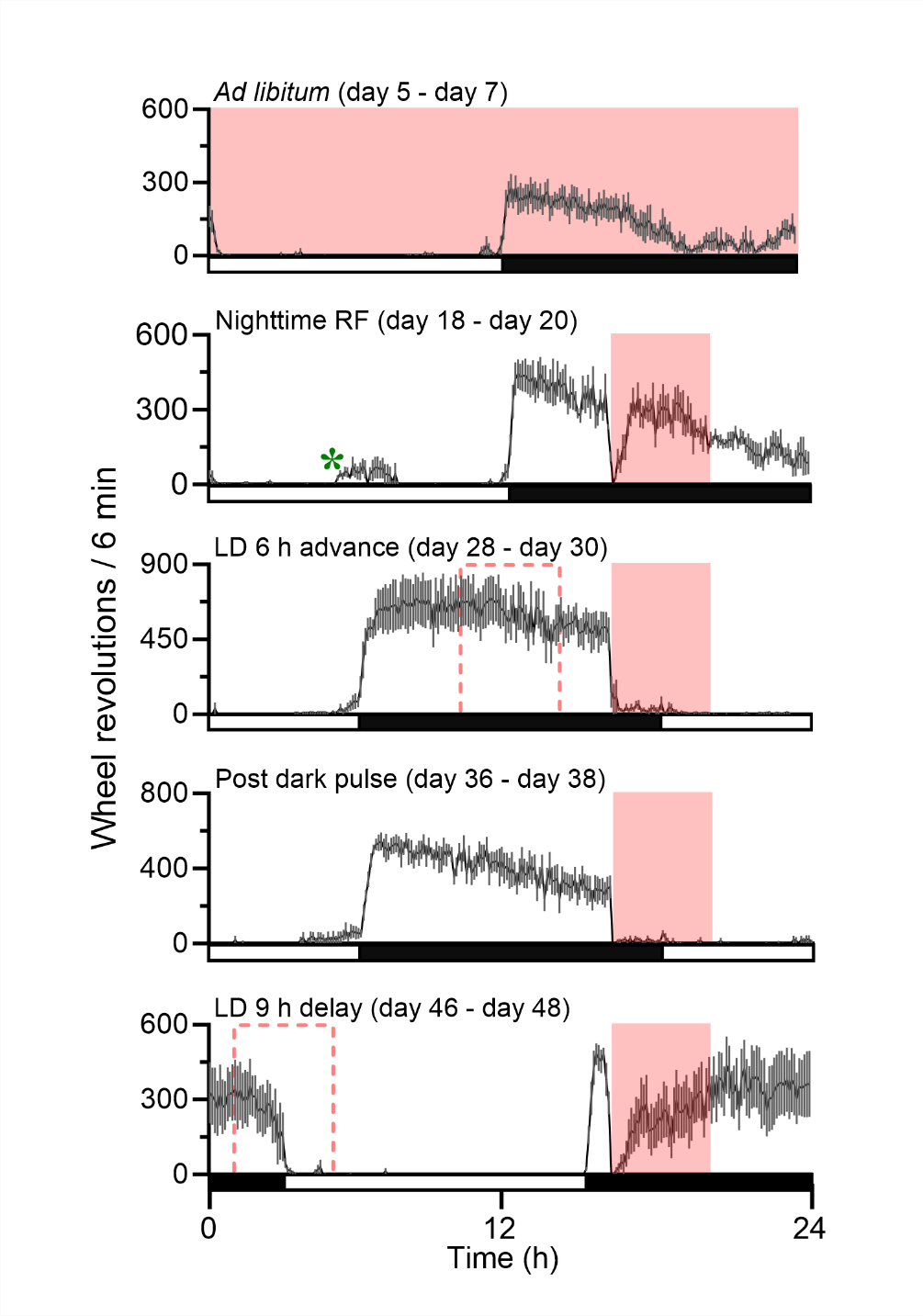
**

**Supplementary Figure 2: Group average 24-h profiles of the wheel running activity.** Related to Figure 1. Group average wheel running activity of 3 days during *ad libitum* (day 5 – day 7), 3 days during night-time restricted feeding (day 18 – day 20), 3 days during the period after the light-dark cycle was advanced (day 28 – day 30), 3 days immediately after the incidental dark pulse (day 36 – day 38), and 3 days during the period after the light-dark cycle was delayed (day 46 – day 48) indicated as blue vertical lines in Figure 1A. The time of food availability is indicated by the solid pink box. The pink dotted box indicates the time of food availability if it were linked to the phase of the light-entrainable oscillator. Data are presented as mean ± SEM. Green asterisk indicates the time of cage change. Activity was temporarily increased after the cage change.

**
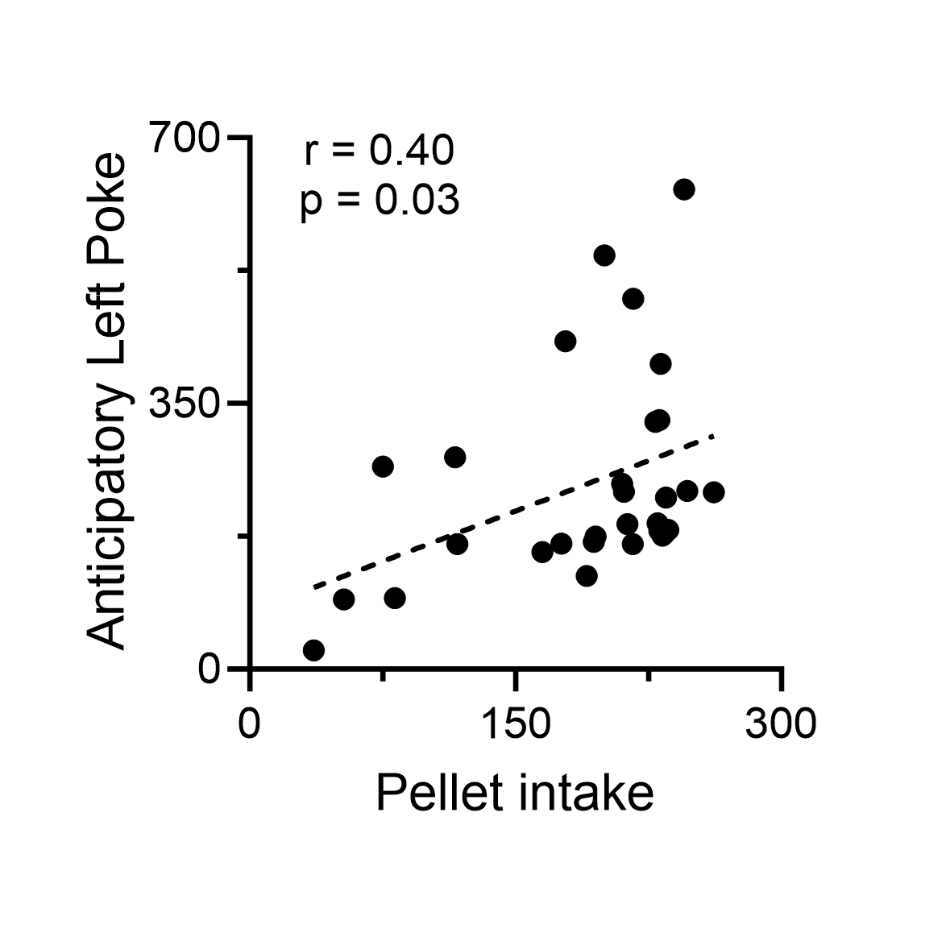
**

**Supplementary Figure 3: Positive correlation between anticipatory nose poking and pellet intake.** Related to Figure 2. The total number of anticipatory nose pokes during the 3 h FAA window and the total pellet intake during 4 h restricted feeding window for the 3 days before and 3 days after the second incidental dark pulse (on day 35) were plotted. Pearson correlation analysis was performed using GraphPad Prism 10.1.2.
